# Comparison of 2 Assays for Diagnosing Rotavirus and Evaluating Vaccine Effectiveness in Children with Gastroenteritis

**DOI:** 10.3201/eid1908.130461

**Published:** 2013-08

**Authors:** Jacqueline E. Tate, Slavica Mijatovic-Rustempasic, Ka Ian Tam, Freda C. Lyde, Daniel C. Payne, Peter Szilagyi, Kathryn Edwards, Mary Allen Staat, Geoffrey A. Weinberg, Caroline B. Hall, James Chappell, Monica McNeal, Jon R. Gentsch, Michael D. Bowen, Umesh D. Parashar

**Affiliations:** Centers for Disease Control and Prevention, Atlanta, Georgia, USA (J.E. Tate, S. Mijatovic-Rustempasic, K.I. Tam, F.C. Lyde, D.C. Payne, J.R. Gentsch, M.D. Bowen, U.D. Parashar);; University of Rochester School of Medicine and Dentistry, Rochester, New York, USA (P. Szilagyi, G.A. Weinberg, C.B. Hall);; Vanderbilt University Medical Center, Nashville, Tennessee, USA (K. Edwards, J. Chappell);; Cincinnati Children's Hospital Medical Center, Cincinnati, Ohio, USA (M.A. Staat, M. McNeal)

**Keywords:** rotavirus, enzyme immunoassay, RT-PCR, viruses, diagnosis, children, acute gastroenteritis

## Abstract

We compared rotavirus detection rates in children with acute gastroenteritis (AGE) and in healthy controls using enzyme immunoassays (EIAs) and semiquantitative real-time reverse transcription PCR (qRT-PCR). We calculated rotavirus vaccine effectiveness using different laboratory-based case definitions to determine which best identified the proportion of disease that was vaccine preventable. Of 648 AGE patients, 158 (24%) were EIA positive, and 157 were also qRT-PCR positive. An additional 65 (10%) were qRT-PCR positive but EIA negative. Of 500 healthy controls, 1 was EIA positive and 24 (5%) were qRT-PCR positive. Rotavirus vaccine was highly effective (84% [95% CI 71%–91%]) in EIA-positive children but offered no significant protection (14% [95% CI −105% to 64%]) in EIA-negative children for whom virus was detected by qRT-PCR alone. Children with rotavirus detected by qRT-PCR but not by EIA were not protected by vaccination, suggesting that rotavirus detected by qRT-PCR alone might not be causally associated with AGE in all patients.

Commercially available enzyme immunoassays (EIAs) traditionally have been used to detect rotavirus in children who have acute gastroenteritis (AGE). The rate of rotavirus detection is higher with EIAs than with conventional and semiquantitative real-time reverse transcription PCRs (qRT-PCRs) (*1*–*6*), but some qRT-PCR–positive samples could represent low-level viral shedding from patients with asymptomatic infections or recently resolved rotavirus infections (*6*). qRT-PCR cycle threshold (C_t_) values correlate inversely with the amount of viral RNA in a specimen. In a study from the United Kingdom, specimens from patients with AGE that tested positive for rotavirus by EIA had significantly lower qRT-PCR C_t_ values (higher viral loads) than did qRT-PCR–positive specimens from patients with AGE that tested negative by EIA and from healthy controls; C_t_ values for the latter 2 groups did not differ (*7*). Another study found that C_t_ values correlated inversely with severity of disease in patients with AGE and EIA-positive specimens (*8*).

Two rotavirus vaccines (RotaTeq [RV5], Merck, West Point, PA, USA, and Rotarix [RV1] GSK Biologicals, Rixensart, Belgium) are recommended for use worldwide (*9*,*10*). These vaccines have demonstrated high efficacy (>85%) against severe rotavirus-associated AGE in the United States and other high-income countries (*11*–*14*). As vaccine use increases, monitoring vaccine impact is important and requires sensitive and specific detection of rotavirus-associated AGE. Several case–control studies of rotavirus vaccine effectiveness have used patients with AGE who test negative for rotavirus by EIA as a comparison group for patients with AGE who test positive by EIA, and concerns have been raised about whether the rotavirus EIA might fail to detect a proportion of true rotavirus cases and thus lead to bias from misclassification of some cases (*11*–*20*). We compared rates of rotavirus detection by EIA and qRT-PCR among children with and without AGE and examined rotavirus vaccine effectiveness against severe cases of rotavirus-associated AGE, as defined by using different combinations of the EIA and qRT-PCR results.

## Methods

### Specimen Collection

Fecal specimens were collected through active surveillance conducted at 3 New Vaccine Surveillance Network sites in the United States (Rochester, NY; Cincinnati, OH; Nashville, TN) year-round during October 2008–October 2009, as described (*18*). In brief, children visiting 1 of the 3 sites who were <5 years of age and had AGE (diarrhea [>3 loose stools in 24 hours] and/or vomiting [>1 episode in 24 hours]) for <10 days and who lived in 1 of the 3 study areas were enrolled, and a fecal specimen was collected. In addition, during this period, fecal specimens were collected from healthy children <5 years of age who resided in 1 of the same 3 study counties and had a well-child visit or an immunization clinic visit at a community medical practice. These healthy children had neither acute respiratory infection symptoms in the 3 days before nor AGE in the 14 days before the recruitment visit. Parents of all enrolled children were interviewed to collect demographic information and disease history.

### Specimen Testing

Fecal specimens were tested for rotavirus by EIA and qRT-PCR. EIA (Premier Rotaclone, Meridian Bioscience, Inc., Cincinnati, OH, USA) testing was done at each study site, and then specimens were frozen and shipped to the Centers for Disease Control and Prevention (CDC, Atlanta, GA, USA) for further testing. All specimens were retested by EIA (Premier Rotaclone, Meridian Bioscience, Inc.) at CDC. If any EIA result, whether obtained at the study site or at CDC, was positive, then the specimen was classified as rotavirus positive. After preparation of a 10% (vol/vol) suspension of each fecal specimen in phosphate-buffered saline, suspensions were clarified by centrifugation at 3,000 rpm for 10 min. A 100-µL volume of clarified supernatant was added to 300 µL of MagNA Pure LC Total Nucleic Acid Isolation Kit Lysis/Binding Buffer (Roche Applied Science, Indianapolis, IN, USA) to lyse the virus and release nucleic acid. RNA was extracted by using the MagNA Pure 96 Cellular RNA Large Volume Kit (Roche Applied Science) and Cellular RNA LV protocol on the automated MagNA Pure 96 instrument (Roche Applied Science) in accordance with the manufacturer’s protocols. The extracted RNA was eluted in 100 µL of elution buffer and stored at −80°C until qRT-PCR testing. RNA was tested for rotavirus by using the NSP3 qRT-PCR designed by Freeman et al. (*3*) and modified to run on an ABI 7500Fast instrument (Applied Biosystems, Foster City, CA, USA) (S. Mijatovic-Rustempasic, KI Tam, TK Kerin, JM Lewis, R Gautam, O Quaye, et al., unpub. data) *21*). C_t_ values that correlated inversely with the amount of virus in the specimen were used as a proxy for viral load. Lower C_t_ values indicated higher viral loads. qRT-PCR was run for 45 cycles and was defined as positive if any virus was detected. Standard viral protein 4 and viral protein 7 sequencing procedures, as described, were attempted for all specimens with virus detected by qRT-PCR to identify vaccine strains (*21*).

### Analysis

We included in the analysis only children who had sufficient sample volumes for complete testing by EIA and qRT-PCR. Healthy children who were enrolled during a vaccination visit and who had a vaccine strain detected in their feces were excluded from the analyses. We compared sociodemographic characteristics, rotavirus detection rates, and C_t_ values by using χ^2^ statistics for categorical variables and Wilcoxon rank-sum tests for continuous variables.

We calculated vaccine effectiveness using the formula (1 – odds ratio for vaccination) ×100 for children >8 months of age. Children are unlikely to receive additional doses of vaccine after 8 months of age. To calculate the adjusted odds ratio, we used unconditional logistic regression and controlled for age at visit, month and year of birth, and month of illness onset. Three laboratory-based rotavirus case definitions were used: EIA positive, qRT-PCR positive, and EIA negative and qRT-PCR positive. Children with AGE who tested negative for rotavirus were used as the control group for the vaccine effectiveness analysis. Three laboratory-based definitions were used for controls: EIA negative, qRT-PCR negative, and EIA negative and qRT-PCR negative. A vaccine dose was considered relevant if it was administered >14 days before enrollment. A child was considered fully vaccinated if he or she had received 3 doses of RV5 >14 days before enrollment. Children whose immunization record could not be obtained were excluded from the vaccine effectiveness analysis. Because RV1 coverage was extremely low during the study period, children who received RV1 also were excluded from the vaccine effectiveness analysis.

## Results

### Study Population

**Figure 1:**
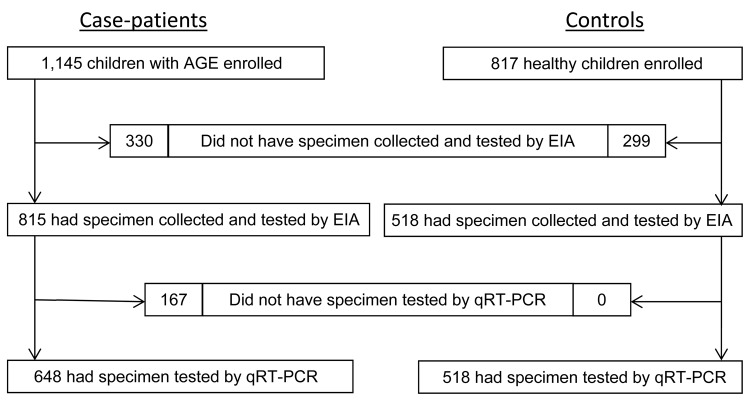
Flowchart of children enrolled in a study of the use of diagnostic assays for rotavirus in children with acute gastroenteritis, 3 New Vaccine Surveillance Network sites (USA), October 2008–October 2009.

Of the 1,145 children whose illnesses met the case definition for AGE during the study period, 815 (71%) had a specimen collected and tested by EIA as part of the surveillance platform (Figure 1). Of these fecal specimens, 648 (80%) were also tested by qRT-PCR, and these 648 children were included in this analysis. Of the 648 specimens tested by both assays, 158 (24%) were positive for rotavirus by EIA. Compared with children whose specimens tested negative by EIA, those whose specimens tested positive for rotavirus by EIA were significantly more likely to be older; be white; have received fewer doses of rotavirus vaccine; have private insurance; live with a child <6 months of age in the household; and have had a specimen collected during January–June, the traditional rotavirus season.

Of the 817 children enrolled as healthy controls, 518 (63%) had a fecal specimen that was tested by EIA and qRT-PCR. Of these, 18 (3%) were enrolled at an immunization visit and had vaccine virus detected in their feces, and they were excluded from further analysis. A total of 500 healthy control children were included in the analysis. Compared with children who had AGE (rotavirus positive or negative by EIA), healthy controls were more likely to be black, fully vaccinated, and have public insurance and less likely to have been breast-fed, attend day care, and have had a specimen collected during January–June. Healthy controls were younger than children positive for rotavirus by EIA and similar in age to children negative for rotavirus by EIA (Table 1).

**Table 1:** Sociodemographic characteristics of patients enrolled in a study of the use of diagnostic assays for rotavirus in children with acute gastroenteritis, 3 New Vaccine Surveillance Network sites (USA), October 2008–October 2009*

Characteristic	Children with AGE				Healthy controls		
	Rotavirus EIA+, n = 158	Rotavirus EIA–, n = 490	p value†		All, n = 500	p value‡	p value§
Median age, mo (IQR)	23 (13–30)	12 (5–23)	<0.001		12 (4–20)	<0.001	0.14
Race			0.04			<0.001	<0.001
White	74 (47)	177 (36)			113 (23)		
Black	45 (28)	196 (40)			293 (59)		
Asian	1 (1)	6 (1)			7 (1)		
Other	38 (24)	111 (23)			84 (17)		
Unknown	0	0			3 (1)		
Hispanic ethnicity	27 (17)	95 (19)	0.56		74 (15)	0.48	0.07
Premature birth	14 (9)	53 (11)	0.47		51 (10)	0.61	0.74
Ever breast-fed	110 (70)	310 (63)	0.16		286 (57)	0.006	0.04
Attended day care	55 (35)	150 (31)	0.28		88 (18)	<0.001	<0.001
No. doses rotavirus vaccine received			<0.001			<0.001	0.02
0	105 (66)	171 (34)			178 (36)		
1	9 (6)	57 (12)			53 (11)		
2	8 (5)	72 (15)			74 (15)		
3	22 (14)	164 (34)			187 (37)		
Ineligible	8 (5)	15 (3)			6 (1)		
Unknown	6 (4)	10 (2)			1 (0)		
Data missing	0	1 (0)			1 (0)		
Insurance status			0.01			<0.001	<0.001
Public	86 (54)	335 (68)			430 (86)		
Private	58 (37)	117 (24)			49 (10)		
Public and private	3 (2)	14 (3)			6 (1)		
None	10 (6)	23 (5)			14 (3)		
Unknown	1 (1)	1 (0)			1 (0)		
Maternal education			0.33			0.07	0.48
Less than high school	44 (28)	134 (27)			141 (28)		
High school	40 (25)	153 (31)			170 (34)		
More than high school	74 (47)	203 (41)			189 (38)		
Age of other child in household							
<6 mo	12 (8)	18 (4)	0.04		26 (5)	0.26	0.24
6–23 mo	23 (15)	50 (10)	0.13		67 (13)	0.71	0.12
2–4 y	51 (32)	145 (30)	0.52		153 (31)	0.69	0.73
<5 y	74 (47)	190 (39)	0.07		214 (43)	0.37	0.20
Season specimen collected			<0.001			<0.001	0.01
January–June	141 (89)	347 (71)			316 (63)		
July–December	17 (11)	143 (29)			183 (37)		
Study site			0.34			0.13	0.72
Nashville, TN	39 (25)	149 (30)			163 (33)		
Rochester, NY	54 (34)	146 (30)			140 (28)		
Cincinnati, OH	65 (41)	195 (40)			197 (39)		

*Values are no. (%) except as indicated. AGE, acute gastroenteritis; EIA, enzyme immunoassay; +, positive; −, negative; IQR, interquartile range. †Children with specimens positive vs. negative for rotavirus by EIA. ‡Children with specimens positive for rotavirus by EIA vs. healthy children. §Children with specimens negative for rotavirus by EIA vs. healthy children.

### Comparison of EIA and qRT-PCR for Rotavirus Detection

#### AGE Cases

**Figure 2:**
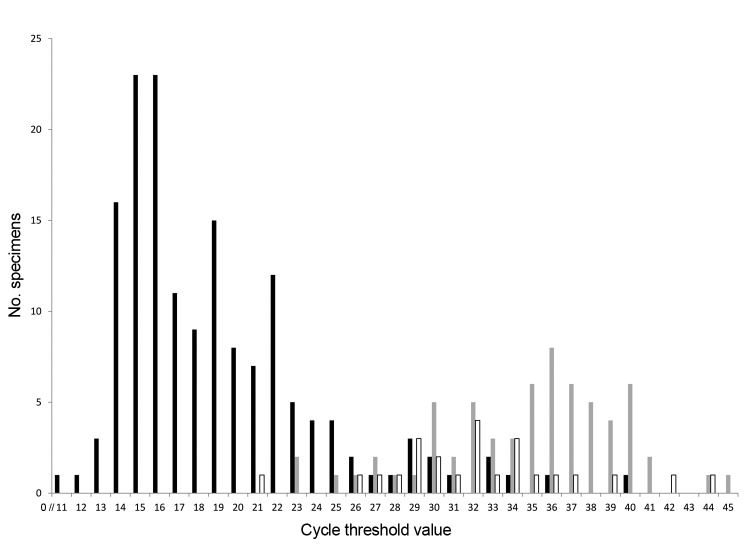
Frequency distribution of C_t_ values for specimens in which rotavirus was detected by qRT-PCR, 3 New Vaccine Surveillance Network sites (USA), October 2008–October 2009. For 1 (1%) acute gastroenteritis EIA+ specimen, 425 (87%) acute gastroenteritis EIA− specimens, and 476 (95%) healthy control specimens, no virus was detected by qRT-PCR. C_t_, cycle threshold; qRT-PCR, semiquantitative reverse transcription PCR; EIA, enzyme immunoassay; +, positive; −, negative. Black bars indicate acute gastroenteritis patients with EIA+ specimens, n = 157; gray bars indicate acute gastroenteritis patients with EIA− specimens, n = 65; white bars indicate healthy controls, n = 24.

For the 158 specimens from children with AGE whose specimens tested positive for rotavirus by EIA, the median C_t_ value was 18 (range 11–40; Figure 2). An RV5 vaccine strain was detected in 1 specimen from an unvaccinated child that tested positive for rotavirus by EIA and had a C_t_ value of 16. No virus was detected by qRT-PCR in 1 (1%) specimen from a child whose fecal sample tested positive by EIA. Specimens from an additional 65 (10%) children were positive by qRT-PCR alone, with a median C_t_ value of 36 (range 23–45), which was significantly higher than the median C_t_ value for EIA-positive children (p<0.001) (Table 2). Rotavirus was detected by qRT-PCR in specimens that were EIA negative and collected during January–June (39 [11%] of 347) and outside the rotavirus season during July–December (26 [18%] of 143).

**Table 2:** Comparison of laboratory results in a study of the use of diagnostic assays for rotavirus in children with AGE, 3 New Vaccine Surveillance Network sites (USA), October 2008–October 2009*

Laboratory result	Children with AGE				Healthy controls		
	Rotavirus EIA+, n = 158	Rotavirus EIA–, n = 490	p value†		All, n = 500	p value‡	p value§
Virus detected by qRT-PCR	157 (99)	65 (13)	<0.001		24 (5)	<0.001	<0.001
Of those with virus detected							
Median C_t_ value (range)	18 (11–40)	36 (23–45)	<0.001		32 (21–44)	<0.001	0.02
G and P type determined	155 (99)	8 (12)	<0.001		12 (50)	<0.001	<0.001
Vaccine strain detected	1 (1)	0	0.52		11 (46)	<0.001	<0.001

*Values are no. (%) except as indicated. AGE, acute gastroenteritis; EIA, enzyme immunoassay; +, positive; −, negative; qRT-PCR, semiquantitative reverse transcription PCR; Ct, cycle threshold.  †Children with specimens that are EIA+ vs. EIA− for rotavirus. ‡Children with specimens EIA+ for rotavirus vs. healthy children. §Children with specimens EIA− for rotavirus vs. healthy children.

No vaccine strains were detected among children with AGE whose specimens tested negative for rotavirus by EIA but positive by qRT-PCR. Wild-type rotavirus strains were detected in 8 (12%) of the 65 specimens with any virus detected, whereas a genotype could not be determined for the remaining 57 (88%) specimens for which virus was detected by qRT-PCR.

#### Healthy Controls

From the 500 healthy control children, 1 specimen tested positive for rotavirus by EIA but not by qRT-PCR. Overall, virus was detected by qRT-PCR in specimens from 24 (5%) healthy children; the median C_t_ value of 32 (range 21–44) was significantly higher than that for EIA-positive children (p<0.001) and significantly lower than that for EIA-negative children (p = 0.02) (Table 2).

Of the 24 healthy controls whose specimens had rotavirus detected by qRT-PCR, 11 (46%) had vaccine virus detected, of which 9 contained an RV5 strain and 2 contained the RV1 strain. Six of these 11 children were unvaccinated, including both children for whom the RV1 strain was detected; 3 had received 1 dose of RV5 (70, 75, and 78 days before enrollment); and 2 had received 2 doses of RV5, with the second dose received 28 and 64 days, respectively, before enrollment. Wild-type virus was detected by qRT-PCR in 13 (3%) of the 500 healthy controls. The median C_t_ values for children with a vaccine virus and a wild-type virus were similar (34 and 30, respectively [p = 0.05]). Wild-type rotavirus was detected by qRT-PCR during the traditional January–June rotavirus season (8 [3%] of 317 specimens) and outside the rotavirus season during July–December (5 [3%] of 183 specimens).

### Vaccine Effectiveness Using Different Definitions for Cases and Controls

Using only the EIA result to define cases and controls among AGE patients >8 months of age, we found that the 3-dose vaccine effectiveness against rotavirus disease that required emergency department care or hospitalization was 84% (95% CI 71%–91%) (Table 3). When cases were defined by using only the qRT-PCR result, the 3-dose vaccine effectiveness estimate decreased slightly to 75% (95% CI 58%–86%), but this estimate did not differ significantly from that estimated by using the EIA result. When cases were restricted to children whose specimens tested negative by EIA but for whom virus was detected at any level by qRT-PCR, the 3-dose vaccine effectiveness estimate was not statistically significant (14% [95% CI −105% to 64%]).

**Table 3:** VE using different case and control definitions in a study of the use of diagnostic assays for rotavirus in children >8 months of age with acute gastroenteritis, 3 New Vaccine Surveillance Network sites (USA), October 2008–October 2009*

Definition, no. doses	No. (%) cases	No. (%) controls	% VE (95% CI)†
EIA+ cases and EIA− controls	128	302	
0	98 (77)	115 (38)	NA
1	6 (5)	15 (5)	51 (−38 to 83)
2	4 (3)	43 (14)	90 (70–97)
3	20 (16)	129 (43)	84 (71–91)
EIA+ case and qRT-PCR− controls	128	266	
0	98 (77)	99 (37)	NA
1	6 (5)	13 (5)	47 (−53 to 82)
2	4 (3)	40 (15)	89 (66–96)
3	20 (16)	114 (43)	83 (68–91)
qRT-PCR+ cases and qRT-PCR− controls	164	266	
0	114 (70)	99 (37)	NA
1	8 (5)	13 (5)	47 (−38 to 80)
2	7 (4)	40 (15)	85 (64–94)
3	35 (21)	114 (43)	75 (58–86)
EIA- and qRT-PCR+ cases vs. EIA- and qRT-PCR- controls	36	266	
0	16 (44)	99 (37)	NA
1	2 (6)	13 (5)	21 (−309 to 80)
2	3 (8)	40 (15)	47 (−108 to 87)
3	15 (42)	114 (43)	14 (−105 to 64)


*VE, vaccine effectiveness; EIA, enzyme immunoassay; +, positive; −, negative; NA, not applicable; qRT-PCR, semiquantitative reverse transcription PCR. †Controlling for age (in months), month and year of birth, and month of illness onset in the analysis.

## Discussion

The rate of rotavirus detection was higher by qRT-PCR than by EIA. Rotavirus was detected by qRT-PCR in fecal specimens from an additional 10% of children with AGE who tested negative by EIA. However, several lines of evidence suggest that rotavirus detected by qRT-PCR alone might not have been the causative agent in some patients with AGE. First, C_t_ values of fecal specimens from children with AGE for whom rotavirus was detected only by qRT-PCR were significantly higher (lower viral loads) than C_t_ values of specimens from children for whom rotavirus was detected by EIA (36 vs. 18). Second, full genotypes could not be determined for most (88%) specimens for which virus was detected by qRT-PCR only, probably because of the low level of viral shedding. Last, rotavirus vaccine showed limited effectiveness against virus identified by qRT-PCR alone, but this result may partially be a function of the small number of cases in this group. In contrast, 3-dose vaccine effectiveness was high (83%–84%) for children whose samples were positive by EIA and comparable to vaccine effectiveness determined by prelicensure trials and other case–control studies in the United States that similarly identified rotavirus-positive cases by EIA (*11*–*13*). These findings, together with the easier implementation of commercial EIAs than qRT-PCRs, support the use of EIA for identifying cases and controls to estimate vaccine effectiveness, even though a few rotavirus infections might be missed by the EIA, particularly in specimens for which C_t_ values are high (low viral loads). qRT-PCR may be useful for identifying cases and controls during vaccine effectiveness studies. However, because such assays also detect low levels of rotavirus circulating in the population but not associated with illness, further work is needed to define a cutoff C_t_ value below which the detected virus is likely to cause illness. This C_t_ value would help to identify the few cases missed by the EIA and exclude cases with low-level background shedding.

Previous studies have compared different methods of detecting rotavirus in fecal specimens. These studies should be directly compared with caution because they used different commercial assays and different PCR techniques; however, trends in patterns of detection can be compared. Similar to researchers in the United Kingdom, we found significantly lower C_t_ values (higher viral loads) in fecal specimens from patients with AGE that tested positive for rotavirus by EIA than in qRT-PCR–positive specimens from patients with AGE whose feces tested negative for rotavirus by EIA or from healthy controls (*7*). Among UK children <5 years of age who had AGE, use of conventional RT-PCR increased the rotavirus detection rate from 17% by ELISA to 54% by PCR (*6*). However, rotavirus also was detected in 23% of healthy controls by PCR, compared with only 1% of those in whom virus was detected by ELISA. In a study in the United States, rotavirus detection rates for patients with AGE were similar between conventional RT-PCR and EIA (53% and 49%, respectively), but in 18% of healthy controls, rotavirus was detected by conventional RT-PCR, whereas no healthy controls were positive by EIA (*4*). We found a much lower rotavirus detection rate among healthy children (5%) than was found in the previous studies in the United States and United Kingdom (18% and 23%, respectively). The low detection rate among healthy controls in our study also might be partially attributable to the eligibility criteria for healthy controls that required a child to be 14 days without AGE before enrollment and the fecal specimen obtained within 5 days enrollment. In addition, unlike the previous studies, our study was conducted after the introduction of rotavirus vaccine into the US immunization program at a time when rotavirus activity had declined substantially (*22*–*24*). The lower detection rate of rotavirus in healthy children in our study may reflect this decrease in rotavirus activity after vaccine introduction; that is, fewer children may have been shedding virus from a previous infection, some may have had an asymptomatic infection, and infected children who had been vaccinated were possibly clearing the virus more quickly.

We detected vaccine virus in 2% of healthy controls, all of whom were either unvaccinated or had not been vaccinated within 4 weeks before illness onset; the source of vaccine virus for these children is unknown. These vaccine strains were detected only by qRT-PCR because no healthy children in whom a vaccine strain was detected were positive for rotavirus by EIA. RV5 virus also was detected in the fecal specimen from 1 unvaccinated child with AGE; the source of vaccine virus for this patient was a recently vaccinated sibling, as described (*21*). This symptomatic patient was positive by both EIA and qRT-PCR.

Our study had some limitations. First, if children were seen for medical care late in their illness or if specimen collection was delayed, rotavirus might have been the cause of symptoms in some children whose specimens tested negative for rotavirus by EIA but showed low levels of qRT-PCR–detected virus. However, 99% of EIA-negative specimens that had low levels of qRT-PCR–detected virus were collected from children within 7 days after they were brought for treatment, and RV5 was not effective against AGE detected by qRT-PCR only, arguing against this possibility. Second, an internal positive control was not used in this study to monitor for false-negative qRT-PCR results possibly resulting from PCR inhibitors in feces that were carried over into the RNA extracts. We believe that the numbers of such samples would have been small because we detected only 1 EIA-positive, qRT-PCR-negative sample in this study. Third, the enrollment of some healthy controls during an immunization visit resulted in oversampling of children shedding vaccine virus. Although we excluded recently vaccinated children in whom vaccine virus was detected, all detected viruses had to be sequenced to identify children who were shedding vaccine virus. However, in a true random sample of healthy children, we would expect some children to be recently vaccinated, so we might have underestimated the proportion of healthy children in whom vaccine virus can be detected. Last, because these data are from an industrialized country in which rotavirus vaccination is routine, our findings might not apply to developing countries where the severity of infection, rates of asymptomatic viral shedding, and performance of the EIA may differ.

In conclusion, our study, which was performed after rotavirus vaccine was introduced, supports the use of EIA for vaccine effectiveness evaluations in patients with AGE, even though EIA may fail to detect some true rotavirus shedding at lower levels. Although qRT-PCR increases the sensitivity of rotavirus detection, some of these cases may be in children with low-level viral shedding from a resolved or asymptomatic wild-type rotavirus infection and not true disease. The use of qRT-PCR with a cutoff C_t_ value should be further examined as a possible diagnostic tool in a range of settings, including in developing countries.
